# miR-125 regulates PI3K/Akt/mTOR signaling pathway in rheumatoid arthritis rats via PARP2

**DOI:** 10.1042/BSR20180890

**Published:** 2019-01-08

**Authors:** Kai Liu, Yingang Zhang, Liang Liu, Qiling Yuan

**Affiliations:** Department of Orthopedic, The First Affliated Hospital of Xi’an JiaoTong University, No. 277, Yanta West Road, Xi’an City, Shanxi Province 710061, China

**Keywords:** miR-125, PARP2, PI3K/Akt/mTOR signaling pathway, rheumatoid arthritis

## Abstract

The present study aimed to explore miR-125 effects on rheumatoid arthritis (RA) development to provide a potential target for RA. Briefly, rat RA model was established (Model group) by injection of Freund’s Complete Adjuvant into the left hind toe. Normal rats injected with saline in the same location were set as Normal group. All rats’ secondary foot swelling degree, polyarthritis index score, spleen and thymus index were measured. Synovial tissues were subjected to Hematoxylin–Eosin (HE) staining and immunohistochemistry. Synovial cells of each group were isolated and named as Normal-C group and Model-C group, respectively. Synovial cells of Model-C group further underwent cotransfection with miR-125 mimics and PARP2-siRNA (mimics+siPARP2 group) or with miR-125 negative control (NC) and PARP2-siRNA NC (NC group). Quantitative reverse transcriptase PCR (qRT-PCR), Western blot, luciferase reporter assay, ELISA, and MTT assay were performed. As a result, compared with Normal group, rats of Model group showed significantly higher secondary foot swelling degree, polyarthritis index score, spleen and thymus index (*P*<0.01). Down-regulated miR-125 and up-regulated PARP2 was found in synovial tissues of Model group when compared with Normal group (*P*<0.01). Synovial tissues of Model-C group exhibited severe hyperplasia and inflammatory cell infiltration. Luciferase reporter assay indicated that PARP2 was directly inhibited by miR-125. Compared with NC group, cells of mimics+siPARP2 group had significantly lower IL-1β, MMP-1 and TIMP-1 levels, absorbance value, and p-PI3K, p-Akt and p-mTOR relative expression (*P*<0.01 or *P*<0.05). Thus, miR-125 might attenuate RA development by regulating PI3K/Akt/mTOR signaling pathway via directly inhibiting PARP2 expression.

## Introduction

Rheumatoid arthritis (RA) was a chronic autoimmune disease. Its incidence was close to 0.5–1% of the entire population [1]. The clinical symptoms of RA were mainly chronic and persistent synovitis, with systemic inflammation and autoantibody production [2]. In most cases, RA did not threaten the lives of patients. However, deformity and functional loss of joint would be caused if without effective treatment. More seriously, the disability caused by RA could seriously affect the quality of life of patients [3].

The etiology and pathogenesis of RA were still unclear, but most researchers considered that multiple factors contributed to the onset of RA, such as genetic, immune, endocrine, environmental, smoking, infection etc [4]. In recent years, the importance of genes in the development and progression of RA had been recognized. As a class of small RNAs, miRNAs played an important role in various diseases. It was reported that abnormal expression of miR-146a, miR-210, and miR-155 was associated with RA. Zhou et al. [5] found in their research that, in patients with RA, miR-146a facilitated a pro-inflammatory phenotype of Tregs via increased STAT1 activation, and contributed thereby to RA pathogenesis. Abdulmaksoud et al. [6] revealed that both miR-210 and miR-155 were closely related with RA disease activity. Serum miR-210 and miR-155 were two independent diagnostic markers for RA and could be served as non-invasive biomarkers for the diagnosis of RA. In Wei et al. [7] research, miR-20a relative expression was significantly decreased. They further indicated that down-regulation of miR-20a could promote STAT3, p-STAT3, and Bcl-2 expression in fibroblast-like synoviocytes.

miR-125 was reported to be involved in many diseases, especially tumors. It was considered as an important tumor suppressor [8,9]. Some studies also demonstrated that miR-125 was involved in some inflammatory reactions. Liu et al. [10] revealed that miR-125 could inhibit goblet cell differentiation in allergic airway inflammation by targetting SPDEF. Duroux-Richard et al. [11] indicated in their article that miR-125 might control monocyte adaptation to inflammation through mitochondrial metabolism and dynamics. However, we realized that the role of miR-125 in RA development and its related mechanisms was researched rarely. So in the present paper, we studied the effect of miR-125 in RA and its related mechanisms. This article might provide a potential therapeutic target for RA prevention and treatment in clinic.

## Methods

### Construction of rat RA model

A total of 20 male Wistar rats (weight: 150 ± 20 g, age: 6 weeks) were purchased from Experimental Animal Center of the Chinese Academy of Sciences. After 1 week of feeding, ten rats were randomly selected, and Freund’s Complete Adjuvant F5881 (Sigma, U.S.A.) at a dose of 0.1 ml/100 g was injected intradermally into the left hind toe for establishing rat RA model. These rats were named as Model group. The remaining ten rats were injected with the same dose of saline in the same location and set as Normal group. All rats were housed 4 weeks after injection [12,13].

In the present study, all animal studies were performed under the supervision and guidelines of the Institutional Animal Care and Use Committee of the Medical School, Xi’an Jiaotong University (Permission Number: SCXK2014-0155, 5 March 2014).

### Measurement of secondary foot swelling degree

Before modeling, the right hind toe volume was measured using a foot swelling gauge. At 1, 2, 3, and 4 weeks after modeling, their right hind toe volume was also measured. The following formula was used to calculate the degree of secondary foot swelling: mean of the toe volume after injection − mean of the toe volume before injection [13].

### Assessment of polyarthritis index score

At 1, 2, 3, and 4 weeks after injection, the degree of systemic joint inflammation of rats was observed, and polyarthritis index score was recorded according to the following scoring guidelines: 0 point, no redness occurred; 1 point, toe joints were swollen; 2 points, toes and toe joints were swollen; 3 points, all paws below the ankle were swollen; 4 points, all paws as well as ankle were swollen. The cumulative score was considered as polyarthritis index score with a maximum of 12 [13].

### Spleen index and thymus index measurements

After 4 weeks, all rats were killed, and spleen and thymus of each rat were obtained and weighed. The spleen and thymus indexes were calculated according to the following formula, respectively: Spleen index = spleen weight/rat body weight. Thymus index = thymus weight/rat body weight [13].

### Hematoxylin–Eosin staining

After the rats were killed, their right hind limbs were removed and fixed with 10% formaldehyde solution for 24 h, followed by decalcification with 10% (mass to volume ratio) EDTA for 30 days. Then these right hind limbs were dehydrated, embedded in paraffin, and sliced. Hematoxylin–Eosin (HE) staining was performed and histopathological changes were observed under a light microscope (Wanheng Precision Instrument Co., Ltd., Shanghai, China) [14].

### Acquisition of rat synovial tissue

All rats were killed and an incision was cut longitudinally in the skin at the midpoint of the rats’ knee joint. The kneecap was seen after muscles were separated. Synovial tissue was then found by continuing downward incision. The synovial membrane and fibrous layer of the joint capsule were separated with an ophthalmic scissors to remove synovial tissues. The other knee joint synovial tissues were collected using the same method [15].

### Isolation and culture of rat synovial cells

The fat fibers of rat synovial tissues were removed under aseptic conditions. D-Hank’s solution without Ca^2+^ and Mg^2+^ was used to wash synovial tissues until these tissues turned white. Then they were washed with Dulbecco’s modified Eagle’s medium (DMEM) (10% FBS) two to three times. An ophthalmic scissors were selected to cut these tissues into small pieces of 1–2 mm^2^ volume in size. These tissue pieces were evenly seeded in culture flasks with DMEM (10% FBS) and incubated in a 37°C, 5% CO_2_ incubator for 8 h. Cells released from synovial tissues were transferred into another flask and cultured with DMEM (10% FBS) in the incubator at the same conditions. Cells isolated from synovial tissues of rats in Normal group were named as Normal-C group, while those released from synovial tissues of rats in Model group were named as Model-C. Cells of each group were subcultured three times [15].

Cells of the two groups were collected in logarithmic growth phase and prepared into cell suspensions using DMEM (10% FBS). They were inoculated in 24-well plates at a density of 1 × 10^5^ cells per well and incubated for 48 h in the incubator at 5% CO_2_, 37°C.

### Immunohistochemistry

Synovial tissues were prepared as slices and five continuous slices of each synovial tissue were selected for immunohistochemical detection. These slices were subjected to xylene dewaxing, gradient alcohol rehydration, and antigen retrieval. They were then placed in goat serum blocking solution for 15 min, followed by three times washing with PBS. Rabbit anti-human PARP2 antibody (1:100, Santa Cruz Biotechnology) was dripped on to these slices for 12 h incubation at 4°C. Secondary antibody (Beijing Zhongshang Jinqiao Biotechnology Co., Ltd., China) was then added for 15 min incubation at 37°C. At last, diaminobenzidine (DAB) chromogenic reaction and Hematoxylin counterstaining for 30 s were performed sequentially. These slices were sealed with neutral rubber after dehydration. Five non-overlapping fields per slice were viewed under a microscope and PARP2 positive cell numbers were counted. Cells with brown particles in the nucleus were PARP2 positive cells [16].

### Quantitative reverse-transcriptase PCR

Rat synovial tissues that had been ground in liquid nitrogen were obtained and their total RNA was extracted using TRIzol reagent (Life Technologies, Gaitherburg, MD). Total RNA in synovial cells of each group was also collected. A total of 5 μl total RNA samples were diluted 20-fold with RNase-free ultrapure water. RNA samples optical density (OD) at 260 nm and 280 nm wavelengths were measured. Samples with OD_260_/OD_280_ ratios between 1.7 and 2.1 were selected for follow-up studies. For each RNA samples, a total of 5 µg was collected for the synthesis of cDNA templates by using PrimeScript Reverse Transcription Reagent Kit (GeneCopoeia Inc., Rockville, MD, U.S.A.) according to the instructions. The PCR amplification reaction was amplified 40 times under the following reaction conditions by using ABI7500 quantitative PCR instrument (Applied Biosystems, U.S.A.): pre-denaturation at 95°C for 10 min, denaturation at 95°C for 10 s, annealing at 60°C for 20 s, extension at 72°C for 34 s. Primer sequences used in the present study were as follows: miR-125, forward, GCUCCCUGAGACCCUAAC, reverse, CAGTGCAGGGTCCGAGGT. U6, forward, CTCGCTTCGGCAGCACATATACT, reverse, ACGCTTCACGAATTTGCGTGTC. PARP2, forward, GTGGAGAAGGATGGTGAGAAAG, reverse, CTCAAGATTCCCACCCAGTTAC. GAPDH, forward, GTCGATGGCTAGTCGTAGCATCGAT, reverse, TGCTAGCTGGCATGCCCGATCGATC.The Tm value for primer of miR-125, U6, PARP2, and GAPDH were 58.1, 58.9, 57.4, and 56.8°C, respectively. The 2^−ΔΔ*C*_T_^ method was chosen to calculate the relative expression of the gene to be tested. miR-125 relative expression was normalized to U6, and PARP2 relative expression was normalized to GAPDH [17].

### Luciferase reporter assay

The binding sites of PARP2 and miR-125 were predicted using TargetScan software. The mutant-type (MT) and wild-type (WT) sequences of the PARP2 and miR-125 binding sites were designed separately, and were integrated into Promega vector. Vectors carrying MT sequences of the binding sites were used to co-transfect normal synovial cells with miR-125 mimics or miR-125 negative controls (NC). They were set as MT+mimics group and MT+NC group, respectively. In addition, miR-125 mimics or miR-125 NCs combined with vectors carrying WT sequences of the binding sites were also used to co-transfect normal synovial cells. These cells were named as WT+mimics group and WT+NC group, respectively. Cells of each group were cultured for 48 h after transfection. Luciferase activity was measured using luciferase kit (Promega Corp., Madison, WI, U.S.A.) [18].

### Cells transfection

**Figure 1:**
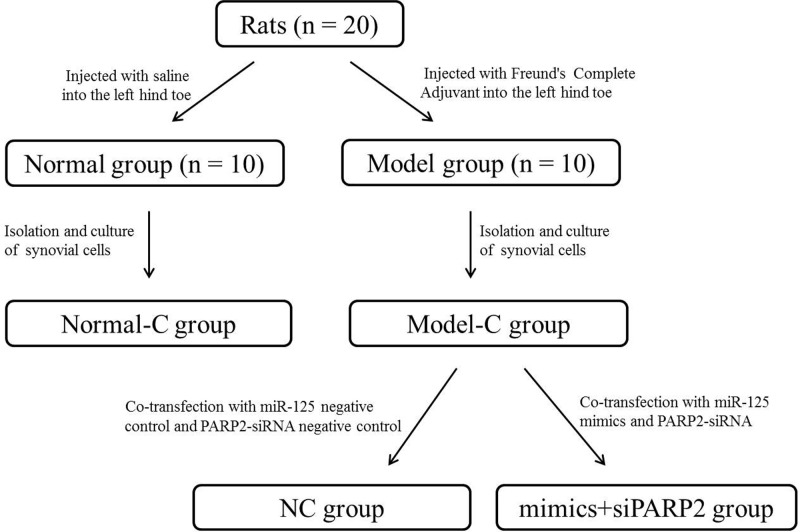
The group flow chart of the entire article

Cells of Model-C group were collected at logarithmic growth phase. They were prepared as single-cell suspension by using DMEM (without FBS), amd were transferred to six-well plates with 1 ml cell suspension per well. Co-transfection with miR-125 mimics and PARP2 siRNA were performed on them. These cells were designated as mimics + siPARP2 group. Similarly, miR-125 NC and PARP2 siRNA NC were simultaneously used to co-transfect cells of Model-C group, and they were named as NC group. All transfectants were purchased from Ruibo Biotechnology Co., Ltd. (Guangzhou, China). The transfection amount of miR-125 mimics, miR-125 NC, PARP2 siRNA, and PARP2 siRNA NC were all 100 nM. All transfections were performed using Lipofectamine 2000 transfection kit (Thermo Fisher Scientific, Waltham, MA, U.S.A.) strictly according to the instructions. Cells that were successfully transfected were selected and cultured in DMEM (10% FBS) in 24-well plates with × 10^5^ cells per well. All 24-well plates were placed in 5% CO_2_, 37°C incubator [19]. In addition, please refer to Figure 1 for a better understanding of the grouping process of the entire article.

### Detection of IL-1β, MMP-1, and TIMP-1 by ELISA

Cells of each group were collected 24 h after transfection. The concentrations of IL-1β, MMP-1, and TIMP-1 in the supernatant were determined by ELISA kit (R&D Systems, Minneapolis, U.S.A.). All operations were performed in strict accordance with the ELISA kit instructions [20].

### MTT analysis

After being cultured for 24, 48, 72, and 96 h, cells of each group were taken out from incubator and 20 μl of MTT solution (5 mg/ml, Sigma–Aldrich, St Louis, MO, U.S.A.) was added into each well for 4 h incubation at 37°C. Residual liquid in each well was removed. A total of 150 μl DMSO was then added into each well. Gentle shaking for 10 min was necessary to promote crystal dissolution. Absorbance value at 495 nm wavelength of each well was determined using an ELISA [21].

### Western blot analysis

Ground rat synovial tissues as well as cells of each group were harvested. Total protein was obtained by using protein lysates. Protein concentration of each sample was determined through BCA protein assay kit (Pierce, Rockford, IL, U.S.A.). A total of 30 mg protein was separated using SDS/polyacrylamide gels at 120 V for 2 h, followed by transferring protein on to PVDF membranes. These PVDF membranes were blocked with 5% skim milk at room temperature for 2 h. Then they were placed in an incubator and incubated with primary antibodies (rabbit anti-human primary antibodies, 1:1000, Cell Signaling, U.S.A.) at 4°C for 12 h. After washing with TBS-tween 20 (TBST) for three times, horseradish peroxidase labeled goat anti-rabbit IgG secondary antibodies (1:5000, Beijing Kangwei Century Biotechnology Co., Ltd., China) were added for 1 h incubation at room temperature. The band densities of specific proteins were quantitated after normalization to the density of GAPDH [17].

### Statistical analysis

In this research, data were analyzed by SPSS17.0. All data were expressed as mean ± S.D. *t* test was used to analyze the comparison between two groups while one-way ANOVA test was used for comparison amongst three or more groups. *P*<0.05 meant that the difference was statistically significant.

## Results

### Rat RA model was successfully constructed

**Figure 2:**
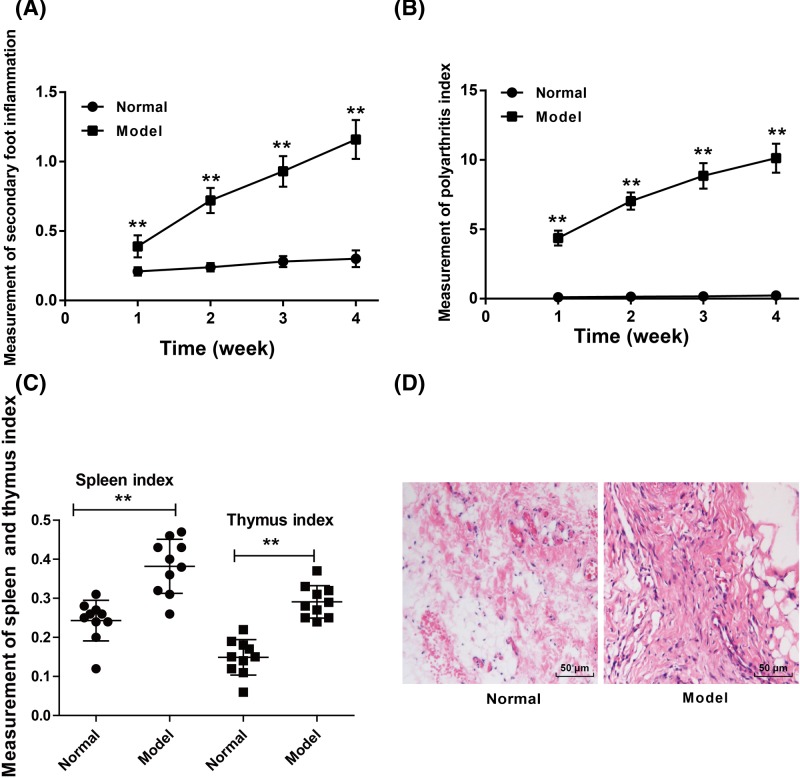
Rat RA model was successfully constructed (**A**) Rats of model group exhibited much higher secondary foot swelling degree than the Normal group. (**B**) Significantly increased polyarthritis index was found in rats of model group when compared with Normal group. (**C**) The spleen and thymus index of rats of model group was much higher than that of Normal group. (**D**) HE staining indicated that the synovial tissues of rats in Normal group were much thinner with one to two layers of synovial cells, whereas the synovial tissues of rats in Model group occurred severe inflammatory cell infiltration with three to five layers of synovial cells. **P<0.01 when compared with Normal group.

For rats of Model group, their secondary foot swelling degree, polyarthritis index, spleen and thymus index were all significantly increased than the rats of Normal group (*P*<0.01) (Figure 2A–C). According to HE staining, we found that the synovial tissues of rats in Normal group were much thinner, which consisted of one to two layers of synovial cells. However, obvious hyperplasia was found in the synovial tissues of rats in Model group. The synovial tissues were consisted of three to five layers of synovial cells and severe inflammatory cell infiltration occurred (Figure 2D). The rat RA model was successfully constructed.

### Down-regulation of miR-125 and up-regulation of PARP2 in rats’ synovial tissues and cells

**Figure 3:**
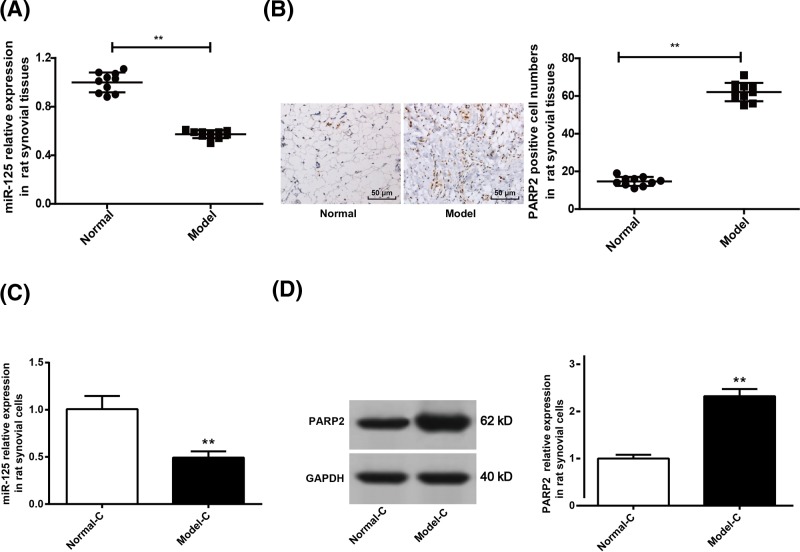
Down-regulation of miR-125 and up-regulation of PARP2 in rats’ synovial tissues and cells (**A**) miR-125 expression in synovial tissues of Model group declined when compared with Normal group. (**B**) Immunohistochemistry results showed that the number of PARP2 positive cells in synovial tissue of Model group was significantly higher than that of Normal group. (**C**) miR-125 expression in synovial cells of Model-C group was markedly lower than that of Normal-C group. (**D**) Significantly increased PARP2 protein expression was observed in synovial cells of Model-C group when compared with Normal-C group. **P<0.01 when compared with Normal group or Normal-C group.

In this research, we investigated the expression of miR-125 and PARP2 in rats’ synovial tissues and cells. The results demonstrated that compared with Normal group, synovial tissues of rats in Model group were with significantly lower miR-125 relative expression and significantly higher PARP2 positive cell numbers (*P*<0.01) (Figure 3A,B). In addition, we also observed that significantly decreased miR-125 relative expression and significantly increased PARP2 positive cell numbers were found in cells of Model-C group when compared with Normal-C group (*P*<0.01) (Figure 3C,D). It indicated that miR-125 was significantly down-regulated while PARP2 was significantly up-regulated in rats’ synovial tissues and cells.

### *PARP2* was a target gene of miR-125

**Figure 4:**
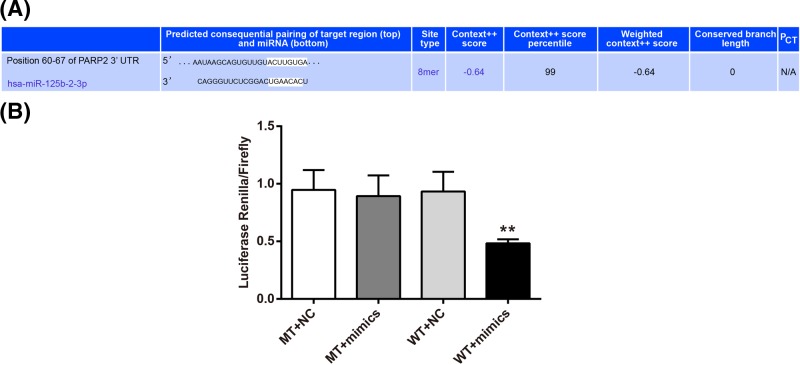
*PARP2* was a target gene of miR-125 (**A**) TargetScan predicted that the 3′-UTR region was the target binding domain of PARP2 and miR-125. (**B**) Significantly decreased luciferase activity intensity was found in WT+mimics group when compared with WT+NC group, indicating that PARP2 was directly inhibited by miR-125. **P<0.01 when compared with WT+NC group.

According to TargetScan online forecast, the 3′-UTR region was the target binding domain of PARP2 and miR-125 (Figure 4A). We further validated the targetting relationship between PARP2 and miR-125 through luciferase reporter activity assay. The results indicated that there was no significant difference in the intensity of luciferase activity between MT+mimics group and MT+NC group. However, compared with WT+NC group, significantly decreased luciferase activity intensity was found in WT + mimics group (*P*<0.01) (Figure 4B).

### Co-transfection of miR-125 mimics and PARP2-siRNA inhibited IL-1β, MMP-1, and TIMP-1 levels in synovial cells of RA rats

**Figure 5:**
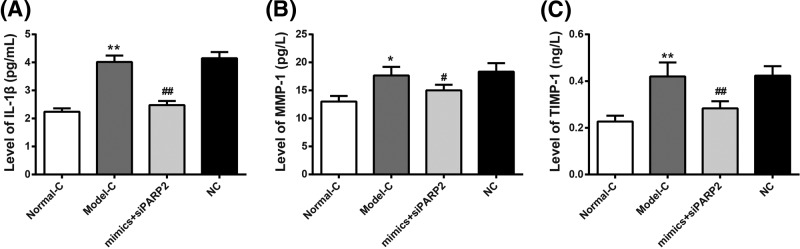
Co-transfection of miR-125 mimics and PARP2-siRNA inhibited IL-1β, MMP-1, and TIMP-1 levels in synovial cells of RA rats (**A**–**C**) According to ELISA, IL-1β, MMP-1, and TIMP-1 levels in synovial cells of Model-C group were significantly higher than those of Normal-C group. Compared with NC group, much decreased IL-1β, MMP-1, and TIMP-1 levels was found in synovial cells of mimics+siPARP2 group. **P<0.01 or *P< 0.05 when compared with Normal-C group. ^##^*P*<0.01 or ^#^*P*<0.05 when compared with NC group.

Detection of ELISA showed that IL-1β, MMP-1, and TIMP-1 levels in synovial cells of Model-C group were significantly higher than those of Normal-C group (*P*<0.01 or *P*<0.05). To further investigate the targetting relationship of miR-125 to PARP2, the present study used miR-125 mimics and PARP2-siRNA or miR-125 NC and PARP2-siRNA NC to co-transfect synovial cells of Model-C group. We realized that compared with NC group, IL-1β, MMP-1, and TIMP-1 levels in synovial cells of mimics+siPARP2 group were significantly lower than those of NC group (*P*<0.01 or *P*<0.05) (Figure 5A–C). The above results suggested that miR-125 might inhibit IL-1β, MMP-1, and TIMP-1 levels in RA rats’ synovial cells by regulating PARP2.

### Co-transfection of miR-125 mimics and PARP2-siRNA inhibited synovial cells proliferation of RA rats

**Figure 6:**
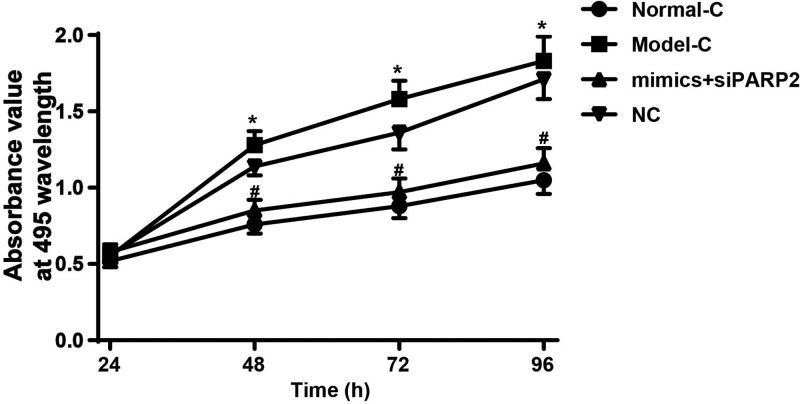
Co-transfection of miR-125 mimics and PARP2-siRNA inhibited synovial cells proliferation of RA rats At 48–96 h, the OD_495_ values of synovial cells in Model-C group were significantly higher than that in Normal-C group. Synovial cells OD_495_ values in mimics+siPARP2 group were significantly decreased when compared with NC group. *P<0.05 when compared with Normal-C group. ^#^*P*<0.05 when compared with NC group.

Cell proliferation was detected using MTT assay. As shown in Figure 6, at 48–96 h, the absorbance values of synovial cells in Model-C group were significantly higher than that in Normal-C group (*P*<0.05). However, after transfection by miR-125 mimics and PARP2-siRNA, synovial cells absorbance values in mimics + siPARP2 group were significantly decreased when compared with NC group (*P*<0.05). We speculated that miR-125 might inhibit synovial cells proliferation by regulating PARP2.

### Co-transfection of miR-125 mimics and PARP2-siRNA inhibited PI3K/Akt/mTOR signaling pathway activity in synovial cells of RA rats

**Figure 7:**
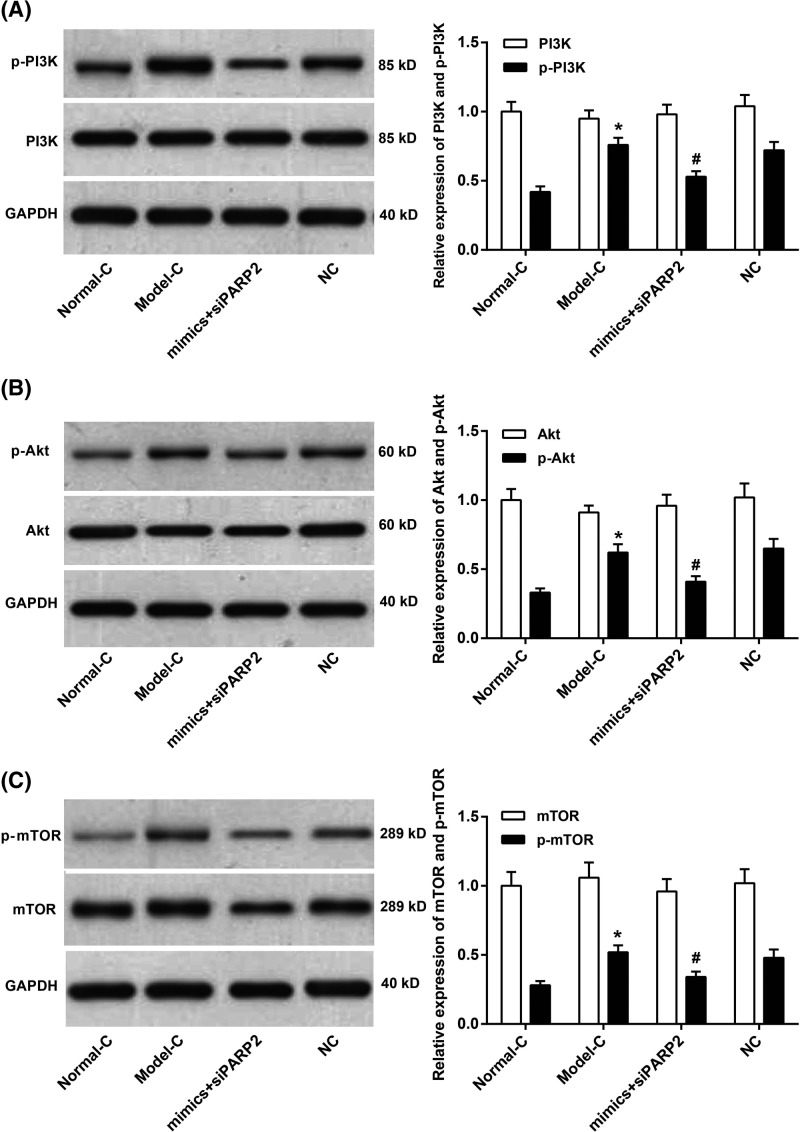
Co-transfection of miR-125 mimics and PARP2-siRNA inhibited PI3K/Akt/mTOR signaling pathway activity in synovial cells of RA rats (**A**–**C**) The relative expression of p-PI3K, p-Akt, and p-mTOR in synovial cells of Model-C group was significantly higher than that of Normal-C group. Compared with NC group, significantly decreased p-PI3K, p-Akt, and p-mTOR relative expression was found in synovial cells of mimics+siPARP2 group. *P<0.05 when compared with Normal-C group. ^#^*P*<0.05 when compared with NC group.

The PI3K/Akt/mTOR signaling pathway was involved in the development and progression of many types of inflammation. This article further studied the activity of PI3K/Akt/mTOR signaling pathway in synovial cells of RA rats. We observed that, amongst synovial cells of Normal-C group, Model-C group, mimics+siPARP2 group, and NC group, the differences in the relative expression of PI3K, Akt, and mTOR were not significant. However, the relative expression of p-PI3K, p-Akt, and p-mTOR in synovial cells of Model-C group was significantly higher than that of Normal-C group (*P*<0.05). Still worth noting was that, compared with NC group, significantly decreased p-PI3K, p-Akt, and p-mTOR relative expression was found in synovial cells of mimics+siPARP2 group (*P*<0.05) (Figure 7A–C). miR-125 might inhibit PI3K/Akt/mTOR signaling pathway activity by regulating PARP2.

## Discussion

RA has a serious adverse effect on the quality of patients’ lives, even life-long disability. Clinically, RA was characterized by abnormal hyperplasia and massive inflammatory cell infiltration [22]. At the same time, RA exhibited multiple and symmetric lesions, which could lead to secondary lesions in the joints of the hands and feet and other joints. Early RA was accompanied by local inflammatory infiltration and synovial hyperplasia of three to five layers. However, advanced RA presented diffuse inflammatory infiltration and more severe synovial hyperplasia [23]. In this study, rat RA model exhibited severe secondary foot swelling degree, polyarthritis index, spleen and thymus index, as well as severe hyperplasia and inflammatory cell infiltration of synovial tissues, which indicated successful construction of rat RA model.

As with many other diseases, the discovery of pathogenesis had important clinical implications for improving the preventive and therapeutic effects of RA. Data from this research demonstrated that, in RA synovial tissues and cells, miR-125 was remarkably down-regulated while PARP2 was significantly up-regulated. Further research suggested that miR-125 might inhibit the development of RA by directly inhibiting PARP2 expression. More and more studies found that miRNAs had important regulatory effects on many human diseases. miR-125, one of the miRNAs, was found to be involved in many human diseases. Especially in several tumors, it exerted as a tumor suppressor. Fan et al. [24] revealed in their research that miR-125 was decreased in tumor tissues of cervical cancer patients, which was negatively correlated with tumor size as well as preoperative metastasis. miR-125 up-regulation could damage the tumor growth, metastasis, and invasion of cervical cancer. Wang et al. demonstrated that miR-125 was down-regulated in human osteosarcoma tissues [25]. The stable overexpression of miR-125 could suppress osteosarcoma cells proliferation, migration, and invasion. They also proved that miR-125 might promote osteosarcoma cell lines chemosensitivity to cisplatin by targetting Bcl-2. However, there were relatively few reports about the effects of miR-125 on inflammation, especially on RA development. Luo et al. [26] reported in their research that decreased miR-125 expression might contribute to the pathogenesis of systemic lupus erythematosus by targetting the expression of ETS1 and STAT3. Murata et al. [27] found that, for patients with systemic lupus erythematosus and osteoarthritis, miR-125 expression level was decreased and plasma concentration of miR-125 could be used as a potential marker for the diagnosis of these inflammatory diseases. Our results illustrated that miR-125 expression was decreased in RA rats’ synovial tissues and cells, and it could directly inhibit PARP2 expression through the combination in the 3′-UTR region. In addition, after co-transfection by miR-125 mimics and PARP2-siRNA, the inflammatory factors levels, such as IL-1β, MMP-1, and TIMP-1, were decreased in RA synovial cells. IL-1β, MMP-1, and TIMP-1 were three important inflammation-promoting factors, which could directly participate in the development of inflammation reactions [28–30]. Filliol et al. [31] observed in their research that, after knockout of PARP2, systemic reduction in NKT cells was found in mice. It further resulted in the reducing hepatocyte death during ConA-mediated liver damage, thereby reducing the risk of hepatitis in rats. Our research found that RA synovial cells proliferation was damaged after they were co-transfected by miR-125 mimics and PARP2-siRNA, confirming that miR-125 could suppress RA development via directly inhibiting PARP2 expression.

Furthermore, the present paper also studied the effects of miR-125 and PARP2 on the activity of PI3K/Akt/mTOR signaling pathway. It could be noticed that PI3K/Akt/mTOR signaling pathway in RA synovial cells was significantly activated, while co-transfection of miR-125 mimics and PARP2-siRNA severely hindered the expression of p-PI3K, p-Akt, and p-mTOR. The PI3K/Akt/mTOR signaling pathway had been reported to be involved in the development of various diseases, especially multiple tumors [25,32,33]. There was still much literature documenting its involvement in inflammatory diseases. Xie et al. [34] pointed out that inactivation of the PI3K/Akt/mTOR signaling pathway could impair renal tubular epithelial inflammation. In Wu et al. [35] study, the inhibitory effect of gambogic acid on RA rats inflammation might be through regulating the PI3K/Akt/mTOR signaling pathway. Results from this paper revealed that miR-125 might inhibit the activity of PI3K/Akt/mTOR signaling pathway via directly inhibiting PARP2 expression.

In short, rat RA model was successfully constructed in the present study. Declined miR-125 and elevated PARP2 in RA tissues and cells was observed. Further *in vitro* studies indicated that miR-125 might attenuate RA development by regulating PI3K/Akt/mTOR signaling pathway via directly inhibiting PARP2 expression. Thus, miR-125 might be used as a potential target for prevention and treatment of RA clinically. Of course, more researches were still needed to confirm this point, and which would be the focus of our future study.

### Ethics approval and consent to participate

The present study was conducted after obtaining approval of the First Afflicted Hospital of Xi’an JiaoTong University’s ethical committee.

## Abbreviations

Bcl-2: B-cell lymphoma-2
DMEM: Dulbecco’s modified Eagle’s medium
ETS1: E26 transformation-specific-1
GAPDH: Glyceraldehyde-3-phosphate dehydrogenase
HE: Hematoxylin–Eosin
IL-1β: Interleukin-1 beta
MMP-1: matrix metallopeptidase 1
MT: mutant-type
NC: negative control
NKT: naturalkiller T
OD: optical density
PARP2: poly ADP-ribose polymerase 2
PI3K/Akt/mTOR: phosphatidylinositol 3-kinase/threonine protein kinase/mammalian target of rapamycin
RA: rheumatoid arthritis
SPDEF: SAM pointed domain containing ets transcription factor
STAT: signal transducer and activator of transcription
TIMP-1: TIMP Metallopeptidase inhibitor 1
Tm: Thrombomodulin
Tregs: Forkhead Box P3
WT: wild-type
